# An Atypical Presentation of Brucellosis in a Patient with Isolated Thrombocytopenia Complicated with Upper Gastrointestinal Tract Bleeding

**DOI:** 10.1155/2012/473784

**Published:** 2012-10-18

**Authors:** Suleyman Baldane, Serdar Sivgin, Tahsin Sezgin Alkan, Fatih Kurnaz, Cigdem Pala, Muzaffer Keklik, Ahmet Karaman, Leylagul Kaynar

**Affiliations:** ^1^Department of Internal Medicine, Faculty of Medicine, Erciyes University, 38039 Kayseri, Turkey; ^2^Department of Hematology, Faculty of Medicine, Erciyes University, 38039 Kayseri, Turkey; ^3^Department of Gastroenterology, Faculty of Medicine, Erciyes University, 38039 Kayseri, Turkey

## Abstract

A 59-year-old female patient was admitted to the emergency service with complaints of hematemesis and melena for the last few days. In laboratory tests, the platelet count was found to be 6 × 10^9^/L. Intravenous or oral corticosteroid treatment was thought to be given for ITP but disclaimed due to upper GIS bleeding. On the 5th day of treatment, *Brucella melitensis* was isolated from blood culture before the results of Wright tube agglutination tests were reported positive as 1 : 80. On the second day of the anti-brucellosis treatment, the thrombocyte count was raised from 6000/mm^3^ to 110000/mm^3^, and on the 3rd day to 225000/mm^3^.

## 1. Introduction

Brucellosis constitutes a major public health and economic problem in many developing countries including Turkey [1]. It is a multisystemic disease, associated with a wide variety of symptoms including hematologic abnormalities such as anemia, thrombocytopenia, pancytopenia, disseminate intravascular coagulation, and leukopenia [2]. *B. melitensis* is the most invasive and causes the most severe disease. Humans are commonly infected through ingestion of raw milk, cheese, or meat or through direct contact with infected animals, products of conception, or animal discharge [3]. We present here *Brucella*-induced thrombocytopenic patient mimicking idiopathic thrombocytopenic purpura (ITP) to emphasize the isolated thrombocytopenia complicated with upper gastrointestinal tract bleeding and the resolution of thrombocyte counts following anti-brucellosis treatment.

## 2. Case

A 59-year-old female patient was admitted to the emergency service of a governmental hospital with complaints of hematemesis and melena for the last few days. She had a history of night sweating, weight loss of up to 10 kilograms in the last 3 months, lumbalgia, fever, and rigors. She was treated empirically for 2 weeks with parenteral sefotaxim, sefazolin sodium and oral nonsteroidal anti-inflammatory drugs, but still continued to suffer. On admission to the emergency service of our university hospital, the vital signs were as follows: body temperature 38.3°C, arterial blood pressure 110/70 mmHg, heart rate 96/min, and respiratory rate 14/min. In physical examination we found that conjunctivas were in pallor and also melena in rectal examination. The respiratory and central nervous system examination was normal. The patient was asked for previous history of any familial disease including coagulopathies, bleeding disorders, and immunological diseases, but did not declare the presence of any. Other physical findings were unremarkable and the patient was hospitalized in the Department of Hematology with probable diagnosis of idiopathic thrombocytopenic purpura (ITP).

In laboratory tests, hemoglobin was 12.6 g/dL, hematocrit 36.8%, white blood cell count 5650 mm^3^ with 52% polymorphs, 44% lymphocytes, 1% eosinophils, and 3% monocytes. The platelet count was found to be 6 × 10^9^/L. In peripheral blood staining evaluation, thrombocytopenia was the same with device results and also atypical lymphoplasmocytoid cells. There was no positive finding for disseminated intravascular coagulation (DIC) like prolongation of prothrombin time (PT), activated partial thromboplastin time (aPTT), and lower levels of fibrinogen. Erythrocyte sedimentation rate was 48 mm/h, prothrombin time, partial thromboplastin time, fibrinogen were normal like renal and liver function tests, sodium, potassium, total protein, and albumin. D-dimer was found highly elevated (5630 *μ*g/L). Urine sediment rate, chest X-ray, and electrocardiogram were normal. Viral antibody screening including cytomegalovirus, Epstein-Barr virus, anti-HIV, and HBsAg were negative and anti-HBs was found to be positive. Serum immunoglobulin G (IgG) was increased (2650 U/mL). No organomegaly was found in abdominal ultrasonographic examination suggesting splenomegaly or hepatomegaly.

As the patient had hematemesis a nasogastric tube was administered. The hemoglobin level decreased to 6.7 g/dL due to massive bleeding. Low platelet and hemoglobin values required both red-blood cell (RBC) and platelet transfusions. A total of 12 packages of RBC and 18 packages of platelet were given to the patient. Bone marrow biopsy was planned but delayed due to the low platelet count (6 × 10^9^). The complete blood count results after infusions were Hb: 9.4 gr/dL and platelet: 28 × 10^9^/L. After platelet infusion, the patient underwent bone marrow biopsy. The biopsy report showed no evidence of increased blast percentage, but the number of megakaryocytes was found to be increased.

**Figure 1:**
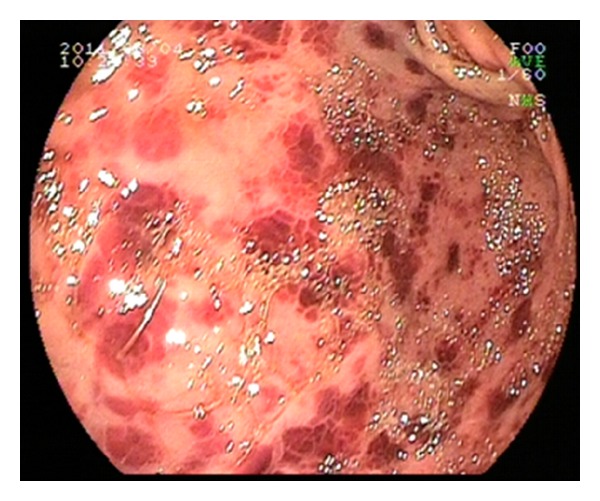
Endoscopic screening of hemorrhagic gastritis.

The blood and urine culture samples were obtained and sent for analysis to related departments. The patient underwent upper gastrointestinal endoscopic screening for upper GIS bleeding. She had disseminated hemorrhagic gastritis (Figure 1) so proton pump inhibitor treatment was continued. Coagulase-negative *Staphylococcus* was isolated from blood cultures on the 5th day of the admission so ampicillin-sulbactam was started at a dose of 3 × 2 grams a day intravenously. In the followup, macroscopic hematuria was present and 12 packages of platelet transfusion with intravenous immunoglobulin (1 mg/kg IV) and 41 packages of fresh-frozen plasma (FFP) were administered due to her probable diagnosis of ITP. Intravenous or oral corticosteroid treatment was considered but not adopted due to upper GIS bleeding. On the 5th day of the treatment, *Brucella mellitensis* was isolated from blood culture before the results of Wright tube agglutination tests were reported positive as 1 : 80 and also B. Coombs test positive as 1 : 160. The patient was consulted with the Department of Infectious Diseases and ampicillin-sulbactam treatment was completed before starting doxycycline 100 mg twice a day orally with rifampicin 600 mg once daily. Echocardiography was performed to exclude the diagnosis of *Brucella* endocarditis and cardiac functions were found to be normal. On the second day of the anti-brucellosis treatment, the thrombocyte count increased from 7000/mm^3^ to 110000/mm^3^, and on the 3rd day to 225000/mm^3^. 

No bleeding remained from nasogastric tube and melena was not present in rectal examination. The patient was discharged from hospital in good health on rifampicin and doxycycline treatment. The duration of anti-brucellosis treatment was planned as 6 weeks. She was in good health on the first visit after discharge and had no side effects of antibiotherapic regimen.

## 3. Discussion

Brucellosis is a systemic disease in which many different organs or systems may be involved. While the disease has been eradicated in many developed countries, it still remains a problem in developing countries. Although the symptoms are nonspecific, the most common complaints include fever, sweats, anorexia, headache, malaise, arthralgia, and lumbalgia. Hematological manifestations of active brucellosis vary from mild anemia and leukopenia to thrombocytopenia and rarely with pancytopenia. The frequency of thrombocytopenia ranges from 3% to 20% [4, 5]. Akdeniz et al. [2] reported a total of 233 patients with brucellosis who had isolated thrombocytopenia lower than 150 × 10^9^/L. These patients had clinically relevant complaints (such as gross hematuria, epistaxis, cutaneous petechiae). Nevertheless, in a study performed by Al-Eissa and Al-Nasser [6] a total of 110 children diagnosed with brucellosis were evaluated. Thrombocytopenia was found in these patients at a percentage of 5%.

Several mechanisms may be involved in the pathogenesis of pancytopenia during the course of brucellosis. These mechanisms involve hypersplenism, bone marrow granulomas, hemophagocytosis and immunologically mediated bone marrow suppression, and disseminated intravascular coagulation [7–9]. The presented case was initially misdiagnosed as ITP but we could not reach a certain decision because corticosteroid administration was contraindicated. Young et al. [10] reported a good response to corticosteroids in the majority of patients diagnosed with brucellosis who had brucellosis-induced thrombocytopenia. Corticosteroid treatment may be beneficial in thrombocytopenias developing due to ITP, infectious-mediated, and immune-mediated because of all their common etiology. The presence of fever may challenge the diagnosis of ITP [11, 12]. Bone marrow biopsy may help to differentiate inadequate production versus excessive destruction/consumption as the predominant cause of thrombocytopenia. In general, a bone marrow biopsy is indicated when a platelet production problem is suspected in order to help delineate the cause of underproduction. In our patient, the history of fever before admittance to the hospital should have alerted the physician to the possibility of diagnosis of infectious disorders rather than autoimmune disorders.

We know that when peripheral destruction or sequestration is suspected, such as in immune thrombocytopenic purpura (ITP), DIC, or thrombotic thrombocytopenic purpura (TTP), a bone marrow biopsy is unlikely to provide useful additional information. However, in elderly patients, patients with simultaneous abnormalities in red and/or white cells, and in patients without a definitive cause, the physicians can elucidate the presence of aprimary marrow disorder, such as myelodysplasia, leukemia, or lymphoma with a bone marrow biopsy. The differential diagnosis of thrombocytopenia is shown in Table 1 [13].

Among the diagnostic procedures of brucellosis, bone marrow cultures were found to be significantly more sensitive compared with blood cultures (92% versus 70%, resp.). Unfortunately, bone marrow culture samples were not obtained due to ongoing bleeding and thrombocytopenia. In a review by Young et al. [10], a total of 41 patients with brucellosis were complicated with thrombocytopenia causing purpura and mucosal bleeding. The most common site of bleeding were as follows: epistaxis (64%), gingival bleeding (44%), and hematuria (64%). In our patient, upper GIS bleeding was present and it was difficult to control the bleeding due to severe thrombocytopenia. In our experience of patients with brucellosis, massive upper GIS bleeding is not an expected type of manifestation for brucellosis. Therefore, firstly, hematological disorders other than brucellosis were thought to be involved. Additional to our patient, data are available presenting brucellosis-induced thrombocytopenic patients receiving intravenous immunoglobulins, and corticosteroids with or without brucellosis treatment [14–16]. Despite the fact that brucellosis is considered to be a benign disorder with favorable outcomes, the treatment strategy for complicated cases is not well established. One of our patient's disadvantages is that the laboratory results of agglutination tests were reported after the results of blood cultures. In routine laboratory evaluations, agglutination tests are usually reported before blood cultures. Therefore, the patient was misdiagnosed for 5 days and was treated with medications out of anti-brucellosis.

Sometimes autoimmune stimulation may cause haemolysis, hemorrhagic purpura, and thrombocyte destruction. Autoimmune thrombocyte destruction may significantly threaten life. The excellent and prompt response to anti-brucellosis treatment in this phenomenon underlines the importance of clinical awareness, especially in endemic areas [15]. Antiplatelet antibodies may cause peripheral immune destruction of platelets but it is difficult to detect these antibodies with the usual tests in *Brucella*-induced thrombocytopenia. A negative assay for antiplatelet antibodies does not exclude the diagnosis of immune thrombocytopenia [5]. Hemorrhages have been reported in 3% to 19% of patients with brucellosis, which is more frequently associated with *B. mellitensis* than with other *Brucella* species [17]. Also, in a study performed by Sari et al. [18], a patient with brucellosis having normal thrombocyte count presented as Coombs-positive autoimmune hemolytic anemia.

This case shows that in regions endemic for brucellosis, bone marrow aspiration and/or biopsy should be planned both for the determination of the cause of pancytopenia or thrombocytopenia. Splenomegaly or hepatomegaly may not be apparent unless the disease reaches further stages of Brucellosis. It may also sometimes be difficult to eliminate any other diseases that may be underlying causes of pancytopenia and thrombocytopenia, even if brucellosis is accurately diagnosed in the patients due to active complications.

## Figures and Tables

**Table 1:** Differential diagnosis of thrombocytopenia.

(1) Pseudothrombocytopenia
(2) Congenital thrombocytopenia

(3) Acquired thrombocytopenia
(a) Platelet sequestration
(i) Hypersplenism
(b) Decreased production
(i) Neoplasia (bone marrow infiltration or cytotoxic drugs)
(ii) Viruses (EBV, CMV, rubella, varicella, parvovirus)
(iii) Megaloblastic anemia
(c) Increased destruction
(i) Immune-mediated (ITP, neonatal alloimmune thrombocytopenia, drug-induced ITP, post transfusion, autoimmune diseases,
lymphoproliferative disorders, HIV, HCV, *Helicobacter pylori* infections, HIT)
(ii) Not immune-mediated (vascular prostheses, DIC, TTP/HUS, HELLP, eclampsia)

Abbreviations: EBV: Epstein-Barr virus; CMV: cytomegalovirus; ITP: idiopathic thrombocytopenic purpura; HIV: human immunodeficiency virus; HCV: hepatitis C virus; HIT: heparin-induced thrombocytopenia; DIC: disseminated intravascular coagulation; TTP: thrombotic thrombocytopenic purpura; HUS: heamolytic uraemic syndrome; HELLP: heamolysis, elevated liver enzymes, low platelets. Adapted from Veneri et al. [13].
